# P-1023. Prevalence and distribution of fungal microorganisms among cutaneous specimens submitted to community microbiology laboratories

**DOI:** 10.1093/ofid/ofae631.1213

**Published:** 2025-01-29

**Authors:** Eugene Yeung

**Affiliations:** University of Ottawa

## Abstract

**Background:**

Not much is known about the local prevalence of cutaneous fungal infections in Canada because these data are not readily available. Knowing the distribution of microorganisms in local settings may help developing guidance for empiric therapy, especially for immunocompromised patients whose cutaneous fungal infections could turn into systemic infections. The current retrospective study aimed to establish the prevalence and distribution of fungal microorganisms among patients who had specimens submitted to community microbiology laboratory for cutaneous fungal culture workups.

**Methods:**

LifeLabs regional microbiology laboratories, connected with 129 collection centers in urban and rural communities in British Columbia, provided reports of isolated fungus from hair, nail, and skin scraping specimens from 1 January 2022 to 31 December 2022. Culture followed by microscopy and/or MALDI-TOF mass spectrometry was routinely used to isolate and identity microorganisms, but nucleic-acid testing was added to help identification if needed.

**Results:**

Among the 20652 cutaneous specimens submitted in a year, 3665 of them were reported to have microorganism growth. The most reported microorganism was *Trichophyton* species (43.8%), followed by *Penicillium* species (5.8%), *Candida albicans* (4.2%), *Aspergillus* species (3.6%), and *Fusarium* species (2.8%). Of note, other than *Trichophyton* species, the other two main dermatophytes *Microsporum* species and *Epidermophyton* species accounted for only 0.2% and 0.1% of the positive specimens, respectively. *Pseudomonas aeruginosa* was incidentally identified in 2.9% of the positive specimens in the fungal culture workups.

**Conclusion:**

*Trichophyton* species was the most reported microorganism among the hair, nail, and skin scrapping specimens submitted for cutaneous fungal culture workups from communities in British Columbia. Pseudomonas infections, which could present as chloronychia, folliculitis, ecthyma gangrenosum, could be considered in the differential diagnoses of cutaneous fungal infections. The current data will help clinicians to determine the empiric therapy needed based on each patient’s clinical situation.

**Disclosures:**

**All Authors**: No reported disclosures

